# Significance of decoy receptor 3 (Dcr3) and external-signal regulated kinase 1/2 (Erk1/2) in gastric cancer

**DOI:** 10.1186/1471-2172-13-28

**Published:** 2012-06-06

**Authors:** Donghai Yang, Xin Fan, Ping Yin, Qiang Wen, Feng Yan, Sibo Yuan, Bin Liu, Guohong Zhuang, Zhongchen Liu

**Affiliations:** 1 Anti-Cancer Research Center, Medical College, Xiamen University, Xiamen, China; 2 Zhongshan Hospital, Xiamen University, Xiamen, China

## Abstract

****Background**:**

Decoy receptor 3 (DcR3), a member of the tumor necrosis factor receptor (TNFR) superfamily, is associated with anti-tumor immunity suppression. It is highly expressed in many tumors, and its expression can be regulated by the MAPK/MEK/ERK signaling pathway. The MAPK/MEK/ERK pathway has been reported to be a regulator in tumor occurrence, development and clonal expansion. External-signal regulated kinase (ERK) is a vital member of this pathway.

****Results**:**

The expression of DcR3 and ERK1/2 in tumor tissues of gastric cancer patients was significantly higher than the non-cancerous group (*P* < 0.05). There was no statistical difference among tumor tissues from patients with different ages or gender, and even of different differentiation (*P* > 0.05). However, in patients with stage I gastric cancer, the DcR3 and ERK1/2 levels were significantly lower than patients with more advanced stages.

****Conclusions**:**

DcR3 and ERK1/2 play a vital role in the development of gastric cancer, and they may be new markers for indicating the efficiency of gastric cancer treatment in the future.

## Background

Decoy receptor 3 (DcR3) is a member of the tumor necrosis factor receptor (TNFR) superfamily. It has been shown to be the decoy receptor for Fas ligand (FasL), LIGHT and TL1A [1-3], also known as TR6 [4]. DcR3 is mostly expressed in tumor cells and competitively inhibits TNF signaling. Overexpression of DcR3 in tumor cells protects them from apoptosis. DcR3 protects tumor cells from immune surveillance as it contributes to the suppression of the host anti-tumor immunity.

DcR3 mRNA and protein are amplified in various malignant tissues, such as lung cancer, colon cancer, gastric cancer, oesophageal carcinoma, pancreas cancer and malignant melanoma [1,5,6]. Wu *et al.*[7] reported that DcR3 could not be detected in non-tumor patients, but could be detected in 98.8% (82/83) of patients with malignant cancers.

This phenomenon demonstrates that the elevated DcR3 expression is significantly correlated with tumorigenesis and tumor progression. Wu *et al.*[8] reported that DcR3 was highly expressed in human gastric cancer (GC), and positively correlated with the development and metastases of gastric lesions. Gastric cancer patients with a high DcR3 expression presented a more advanced pN2-3 disease than those with a low DcR3 expression.

The DcR3 for FasL may be involved in the progression of gastric cancer. Further evaluation of the possible roles of DcR3 and the regulation of DcR3 expression in malignant cells is very important for the development of new strategies for controlling the growth of malignant cells that escape the host immune surveillance.

At the early stage, the MAPK/ERK pathway is activated in tumors, which is a notable sign for many kinds of cancers in humans. Recently, several lines of evidence suggested that ERK could be a parameter for predicting the prognosis of various cancers such as breast cancer, colon cancer, pancreas cancer and cholangiocarcinoma. Wang *et al.*[9] reported that the expression of ERK1/2 was high in cholangiocarcinoma, which correlated with the TNM stages.

Kim *et al.*[10] found that LPS induced DcR3 release in human intestinal epithelial cells (IEC), which appeared to be via the activation of mitogen-activated protein kinases (MAPK), such as extracellular signal-regulated kinase 1 and 2 (ERK1/2) and c-Jun NH2-terminal protein kinase (JNK). LPS-induced DcR3 release in SW480 cells was abolished by ERK1/2 and JNK inhibitors. Furthermore, Yang *et al.*[11] reported that 3 g/ml DcR3 markedly induced the phosphorylation of ERK and p38.

In accordance with these reports, we propose that DcR3 and ERK1/2 are closely related. The DcR3 genes closely correlated with the occurrence and development of many kinds of tumors. In this study we investigated the expression level and location of DcR3 and ERK1/2 in gastric cancer patients of different ages, gender, stage and differentiation, to explore the relationship between DcR3/ERK1/2 and gastric cancer occurrence and development. Furthermore, we provide suggestions for clinical diagnosis of gastric cancers.

## Methods

### **Clinical samples**

Tumors were from gastric cancer patients who were undergone endoscopic biopsy or curative operations in Zhongshan hospital affiliated with Xiamen university. Tumor tissues from which DNA and protein were isolated were from fresh specimen of resection surgery. All samples were obtained with patient consent and approval of the Committee on Medical Ethics of Zhongshan Hospital Xiamen University.

### **Mice**

All the experiments were performed using 6–8 weeks male nude mice purchased from Model Animal Research Center of Medical College Xiamen University. All the animals were housed under specific pathogen free conditions with constant access to water and chow. All experimental procedures were carried out following the approval of the Animal Care and Use Committee of Xiamen University.

### **Cell culture**

The human gastric cancer cell line BGC823 was maintained in our laboratory, which was cultured in flasks with Dulbecco’s modified Eagle’s medium (DMEM) supplemented with 10% fetal bovine serum (FBS), and 1% penicillin-streptomycin at 37°C in an humidified atmosphere of 5% CO2.

### **Reagents**

DMEM, FBS and penicillin-streptomycin were purchased from Hyclone Corporation (Utah, USA). Hematoxylin-and-eosin (H&E) assay kit were purchased from Chemicon International, Inc (Temecula, CA, USA). ERK1/2 and DcR3 Abs were purchased from Santa Cruz Bio technology. RT-RCR reagents were purchased from TaKaRa (Dalian, China). The primer sequence was synthesized from Sangon (Shanghai, China).

### **In vivo animal tumor models**

Tumors were generated in male nude mice by intramuscular (i.m.) injection of BGC823 cells (1.0 × 105 cells in 100μL PBS) into the right flank. Tumor measurements were converted to tumor volume (V) by the formula (L × W2 × 0.52), where L and W are the length and width, respectively. Measurements were made with a vernier caliper. All tumor-bearing mice were divided randomly into groups (2 mice/group).

### **RT-PCR and Western blotting analysis for examining the expression of DcR3/ERK**

Total RNA of DcR3 and ERK1/2 was extracted from stimulated cells using Trizol. To measure ERK/DcR3 gene copy number, DNA from fresh tumor samples was analyzed with RT-PCR. The upstream and downstream primers for ERK1 mRNA were 5'-CCTGCTCATCAACACCACC-3’ and 5'-CGTAGCCACATACTCCGTCA-3’, and for ERK2 mRNA were 5'-TCTTCC AGCCCTCCTTCCTG-3' and 5'-CGTTTCTGCGCCGTTAGGT-3'. The samples were denatured at 95°C for 4 min, followed by 35 cycles of amplification (95°C, 45 sec; 55°C, 45 sec; and 72°C, 60 sec). The products were 300-bp and 400-bp fragments respectively. For DcR3 mRNA, the upstream primer was 5'- GCAAAGCCAAGGATTCCCCCTG -3’ and the downstream primer was 5'-GGCACTGCTCTGAGCTGGAGCTG-3’. The samples were denatured at 95°C for 4 min, followed by 30 cycles of amplification (95°C, 45 sec; 55°C, 45 sec; and 72°C, 60 sec). The product was a 921-bp fragment.

BGC823 cells were harvested and lysed in cell lysis buffer (1% (v/v) Nonidet P-40, 150 mM NaCl, 50 mM Tris–HCl (pH 8), 1 mM PMSF, 2 g/ml aprotinin, and 2 g/ml leupeptin) which could release DcR3 and ERK1/2 proteins. Twenty-five micrograms of total lysate was fractionated by SDS-PAGE and subjected to Western blotting analysis using the anti-ERK or anti-DcR3 mAb (clone 3 H5).

### **Western blotting and ELISA assay for examining the expression of DcR3/ERK after inhibitors treatment**

BGC823 cells culture supernatants were collected at various intervals, and levels of U0126, PD98059, APDC, MEK1/2 and ERK1/2 interferences were quantified using commercial ELISA kits, according to the vendor’s instructions. Cells treated with ERK1/2 shRNA (5:2, 6:2), U0126/PD98059 (0 μmol/L, 5 μmol/L, 10 μmol/L, 20 μmol/L, 40 μmol/L) and APDC (0 μmol/L, 10 μmol/L, 20 μmol/L, 40 μmol/L, 80 μmol/L), respectively. After a 5- or 7-day incubation, cells were subjected to the cytokines as indicated assay. Twenty-five micrograms of total lysate was fractionated by SDS-PAGE and subjected to Western blotting analysis using the anti-ERK or anti-DcR3 mAb (clone 3 H5).

### **Immunohistochemistry analysis for the expression of DcR3 and ERK1/2**

Tumor tissues were fixed in a formaldehyde medium and embedded in paraffin. Sections 6 mm thick were mounted on glass slides pretreated with 0.1% poly-L-Lysine. They were then deparaffinaged in xy-lene, dehydrated in graded ethanol and soaked in 3% H_2_O_2_ for 10 min to eliminate endogenous peroxidase activity. Next, the slides were submerged in citrate buffer (pH 6.0) and boiled at 92–98°C in a microwave oven for 10 min. Subsequently, they were rinsed 3 times with PBS for 10 min each and blocked with 10% normal goat serum in PBS for 1 hr at room temperature. The slides were then reacted with the affinity-purified rabbit anti-TR6 Ab (1.67 g/ml) at room temperature for 2 hr. After washing, the slides were incubated with biotinylated goat-anti-rabbit antibody for 10 min. TR6 signal was revealed by streptavidin-peroxidase using DAB as a substrate according to instructions from the Histostain-Plus kit (Zymed Laboratories, South San Francisco, CA). DcR3 signals were revealed in brown. Finally, the slides were counterstained with hematoxylin and sealed with Aqueous Mounting Media (Zymed).

#### **Statistical analysis**

Data were presented as mean ± SD. The significance of the difference between the groups was assessed by Student`s two-tailed t-test. Probability value of less than 0.05 was considered significant. All means were calculated from at least three independent experiments.

## Results

### **Cancer patients have elevated DcR3 levels**

**Figure 1:**
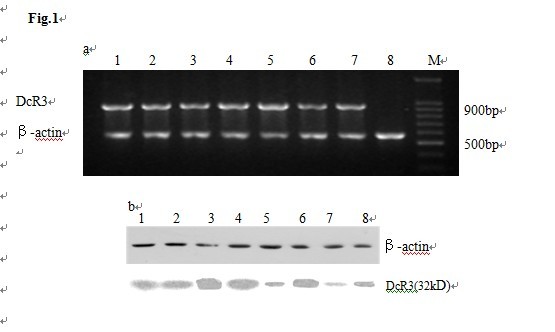
**The expression of DcR3 in gastric cancer and non-cancerous tissues analyzed by RT-PCR (Figure**1a**) and Western blotting (Figure**1b**). a**: Lane M, DNA markers; lanes1-7, tumor tissues; lane 8, non-cancerous tissues; DcR3 mRNA was positive in 36 of 50 tumor tissues (1–7) and negative in non-cancerous tissues (8). **b**: lanes1-7, tumor tissues; lane 8, non-cancerous tissues. DcR3 protein was positive in 37 of 50 samples of tumor tissue (1–7), which only 3 of 50 in non-cancerous tissues (8).

To study the correlation between DcR3 expression and tumor occurrence and development, tumors from 50 gastric cancer patients were collected and tested for DcR3 mRNA and protein levels. As shown in Figure 1a, the 921 bp DcR3 bands were generated by RT-PCR from the tumor tissues of most patients (36/50), compared with the non-cancerous tissues (2/50) from the same organs of these patients. These results demonstrate that DcR3 levels were significantly increased (eight randomly picked clinical samples are shown in Figure 1a). To further confirm our results, the DcR3 protein levels were examined by western blotting. The results demonstrate that DcR3 protein could be detected in most cancer patients; DcR3 protein was detected in 74% (37/50) of the tumor tissues, but only in 6% (3/50) of the non-cancerous tissues (Table 1 and Figure 1b; eight randomly picked clinical samples are shown in Figure 1b).

**Table 1:** Analysis of gastric cancer patients with DcR3 and ERK1/2 expression in tumor and normal tissues

	**Cases**	**Positive number**			**Positive rate (%)**			
Tumor	50	mRNA	Protein	+/+	mRNA	Protein	+/+	
DcR3		36	37	28	72.0	74.0	56.0	
ERK1		37	42	36	74.0	84.0	72.0	*P* > 0.05
ERK2		32	37	27	64.0	74.0	54.0	
Non-cancerous	50							
DcR3		2	3	0	4.0	6.0	0	
ERK1		6	5	1	12.0	15.0	2.0	
ERK2		9	10	3	18.0	20.0	6.0	

### **Expression of ERK1/2 in Clinical samples**

**Figure 2:**
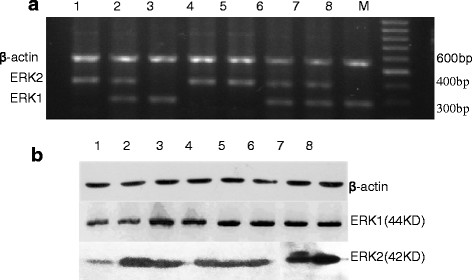
**The expression of ERK1/2 in gastric cancer and non-cancerous tissues analyzed by RT-PCR (Figure **2a**) and Western blotting (Figure **2b**). a**: Lane M, DNA markers; lanes1-7, tumor tissues; lane 8, non-cancerous tissues; ERK1 and ERK2 mRNA were positive in tumor tissues. **b**: ERK1 and ERK2 protein were positive in tumor tissues (1–8).

To identify the signaling molecules related to DcR3, the expression levels of ERK1/2, JNK and p38 were tested. We found that only ERK1/2 could be detected in tumor samples (data not shown for JNK and p38). ERK1 mRNA was detected in 74% of the samples (37/50), and ERK2 mRNA was detected in 64% of the samples (32/50; Table 1 and Figure 2a). At the protein level, ERK1 was detected in 84% (42/50) and ERK2 in 74% (37/50) of the tumor tissues (Table 1 and Figure 2b). These results indicate that patients with gastric cancer tumors have a higher ERK1/2-positive occurrence compared with the non-cancerous tissues (*P* < 0.05). There was a positive correlation between the mRNA and protein levels, as in 42 of 50 tumor tissues the ERK1 protein level was elevated, of which 36 cases were consistent with the mRNA expression. In contrast, ERK1 protein expression was detected only in five normal tissues. When examining ERK2, 37 of 50 cases of tumor tissues were positive for ERK2 protein expression, of which 27 cases were consistent with the ERK2 mRNA expression, while only ten cases showed positive protein expression in normal tissues. The eight randomly picked samples are shown in Figure 2.

### **Location of ERK1/2 in gastric cancer patients**

**Figure 3:**
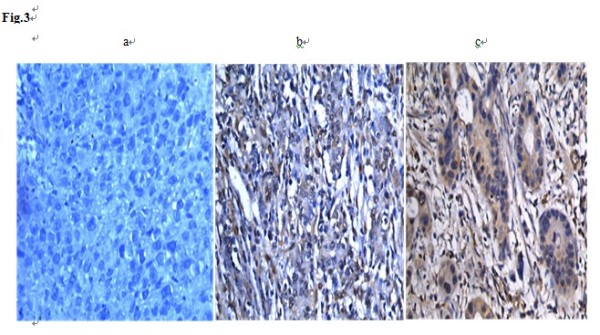
**The location of ERK1/2 protein in gastric cancer tissues analyzed by immunohistochemistry. A**: Control, **B**: ERK1, C: ERK2.

To examine ERK1/2 distribution, tumors from 50 patients were tested by immunohistochemistry. The results show that ERK1/2 were expressed in tumor tissues from most of the patients. ERK1 expression was found in the cytoplasm. ERK2 positive expression was found in some tumor cells (Figure 3).

### **DcR3 and ERK1/2 levels correlate with tumor invasion but not with age, gender or differentiation**

The positive incidence of DcR3 mRNA in tumor tissues of gastric cancer patients was 72.0% (36/50), and of DcR3 protein 74.0% (37/50), which was significantly higher than in non-cancerous tissues showing only 4% (2/50) and 6% (3/50), respectively (*P* < 0.05; Table 1). The positive occurrence of ERK1/2 mRNA expression was 74.0% (37/50) and 64.0% (32/50), and of protein expression 84.0% (42/50) and 74.0% (37/50). Both of them were significantly higher than the non-cancerous tissues, showing 12.0% (6/50) and 18.0% (9/50), and 10.0% (5/50) and 20.0% (10/50), respectively (Table 1). These results suggest that DcR3 and ERK1/2 expression levels correlated with tumor occurrence and development.

As shown in Tables 2, 3 and 4, there was no significant difference between tumor tissues from different ages, gender groups and patients with different differentiation stages of gastric cancer (*P* > 0.05). However, DcR3 and ERK1/2 expression levels were significantly high in TNM stage II-IV (Table 5). Thus, the expressions of DcR3 and ERK1/2 correlated with tumor invasion and TNM stage, but not with age, gender or differentiation.

**Table 2:** Correlation of expression of DcR3 and ERK1/2 with age in gastric cancer patients

	**Cases**	**Positive number**			**Positive rate (%)**			
≥50	32	mRNA	Protein	+/+	mRNA	Protein	+/+	
DcR3		25	28	21	78.1	87.5	65.6	
ERK1		28	31	26	87.5	96.9	81.2	*P* > 0.05
ERK2		20	22	16	62.5	68.8	50.0	
<50	18							
DcR3		11	9	5	61.1	50.0	27.8	
ERK1		13	11	5	72.2	61.1	27.8	
ERK2		12	15	6	66.7	83.3	33.3	

**Table 3:** Correlation of expression of DcR3 and ERK1/2 with gender in gastric cancer patients

	**Cases**	**Positive number**			**Positive rate (%)**			
Male	39	mRNA	Protein	+/+	mRNA	Protein	+/+	
DcR3		29	29	24	74.4	74.4	61.5	
ERK1		32	35	28	82.1	89.7	71.8	*P* > 0.05
ERK2		26	30	23	66.7	76.9	59.0	
Female	11							
DcR3		7	8	5	63.6	72.7	45.6	
ERK1		9	7	6	81.8	63.6	54.5	
ERK2		6	7	4	54.6	63.6	36.4	

**Table 4:** Correlation of expression of DcR3 and ERK1/2 with tumor differentiation in gastric cancer patients

	**Cases**	**Positive number**			**Positive rate (%)**			
**Well**	9	mRNA	Protein	+/+	mRNA	Protein	+/+	
DcR3		7	7	5	77.8	77.8	55.6	
ERK1		7	9	6	77.8	100.0	66.7	*P* > 0.05
ERK2		6	7	5	66.7	77.8	55.6	
**Moderately**	28							
DcR3		21	23	20	75.0	82.1	71.4	
ERK1		24	24	22	85.7	85.7	78.6	
ERK2		19	24	17	67.9	85.7	60.7	
**Low**	13							
DcR3		8	7	5	61.5	53.9	38.5	
ERK1		10	9	8	76.9	69.2	61.5	
ERK2		7	6	5	53.9	46.2	38.5	

**Table 5:** Correlation of expression of DcR3 and ERK1/2 with pathologic stages of tumour in gastric cancer patients

**TNM stage**	**Cases**	**Positive number**			**Positive rate (%)**			
I	6	mRNA	Protein	+/+	mRNA	Protein	+/+	
DcR3		1	1	1	16.7	16.7	16.7	
ERK1		2	2	1	33.3	33.3	16.7	*P* > 0.05
ERK2		1	1	0	16.7	16.7	0.0	
II	17							
DcR3		12	13	10	70.6	76.5	58.8	
ERK1		14	14	11	82.4	82.4	64.7	*P* > 0.05
ERK2		10	12	9	58.8	70.6	52.9	
III	25							
DcR3		21	21	21	84.0	84.0	84.0	
ERK1		23	24	22	92.0	96.0	88.0	*P* > 0.05
ERK2		20	23	18	80.0	92.0	72.0	
IV	2							
DcR3		2	2	2	100.0	100.0	100.0	
ERK1		2	2	2	100.0	100.0	100.0	*P* > 0.05
ERK2		1	1	1	50.0	50.0	50.0	

### **Expression levels of DcR3 and ERK1/2 are amplified in animal models**

**Figure 4:**
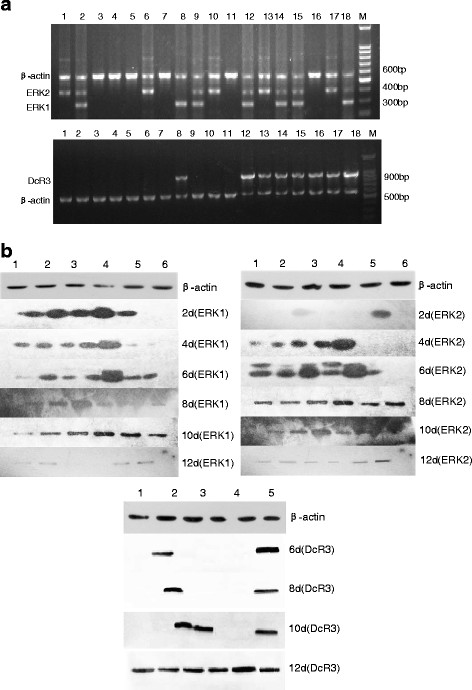
**The expression of DcR3 and ERK1/2 in mouse models analyzed by RT-PCR (Figure **4a**) and Western blotting (Figure **4b**). a**: Lane M, DNA marker; lanes1-6, hearts, livers, spleens, lungs, kidneys and tumor tissues of mouse models on the fourth day; lanes7-12, which on the eighth day ; lanes13-18, on the twelfth day; ERK1 mRNA was positive in lane2,8,9,12,14,15,18, ERK2 mRNA was positive in lane1,2,6,9,10,12,13,14,15,17,18. DcR3 mRNA was detected in lane8, 12, 13, 14, 15, 16, 17, 18. **b**: lane 1, heart; lane2, liver; lane3, spleen; lane4,lung; lane5, kidney; lane6, tumor tissues; The expression of ERK1 protein increased in tumor tissues as time went on, in heart, liver and kidney persisted for 12 days, The expression of ERK2 could be detected in spleen and tumor from the second day which was positive in tumor tissues till the fourth day and all of five organs to the twelfth day. The expression of DcR3 protein was positive in tumor tissues and liver on the sixth day and in spleen on the tenth day, which was negative in heart, lung, kidney until the twelfth day.

As shown in Table 6, after injecting BGC823 cells into the right flank of nude mice, the tumors grew each day. RT-PCR was used to test DcR3 and ERK1/2 mRNA levels in the tumors and western blot analysis was used to examine protein levels. In gastric cancer animal models, DcR3, ERK1 and ERK2 were detected at 921 bp, 300 bp and 400 bp, respectively. In these tumor tissues DcR3 mRNA was detected from day six, peaked on day 10, and remained detected on day 12. DcR3 was also detected from day six to day 12 in liver, while it was only detected on day 12 in heart and lung (Figure 4a). ERK1/2 mRNA expressions were detected from day four in tumor tissues, and ERK1 mRNA peaked on day 10 (Figure 4a and Table 7).

**Table 6:** **Weight of BGC823 tumor in nude mouse (*x*** **±** ***s, n*** **= 6)**

**Group**	**Δ weight/ mg**					
	**2 d**	**4d**	**6d**	**8d**	**10 d**	**12 d**
Normal	—	—	—	—	—	—
Model	0. 47 ±0. 17 ^△^	0. 77 ±0. 28 ^△^	1. 01 ±0. 39 ^△^	1. 43 ±0. 79 ^△^	1. 65 ±0. 68 ^△^	1. 46 ±0. 66^△^

Note: ^△^*P* < 0.01 *vs* normal; **P < 0.01 *vs* model.

**Table 7:** Expression of DcR3 and ERK1/2 mRNA in tumor tissues of animal models analyzed by RT-PCR

	**2d**	**4d**	**6d**	**8d**	**10d**	**12d**
DcR3	—	—	+	+	++	++
ERK1	—	+	+	+	++	++
ERK2	—	+	+	+	+	+

DcR3 protein was detected on day six in tumor tissues and liver, and the expression remained until day 12. DcR3 protein was detected on day 10 in spleen and by day 12 in heart, kidney and lung. ERK1 protein was detected on day four in tumor tissues, and continued to increase each day. ERK1 protein remained at a stable level in heart, liver and kidney, but it decreased in lung and spleen on day 10, after reaching the peak. ERK2 was detected on day two in spleen and tumor tissues. From day four, ERK2 protein was detected in all six samples, and continued to increase till day 12. These results suggest that ERK1 and ERK2 might have different effects on tumor occurrence, development and clonal expansion.

### **DcR3 expression decreased after inhibiting the expression or phosphorylation of ERK1/2 in BGC823 cells**

**Figure 5:**
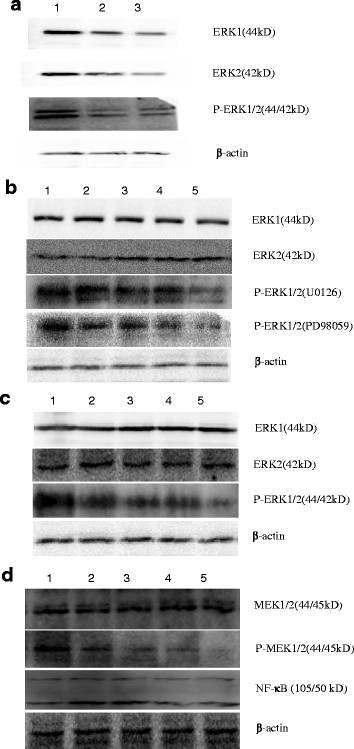
**The expression of ERK1/2 and P-ERK1/2 in BGC823 cell line with plasmid interference and inhibitor detected by Western blotting. a**: The expression levels of ERK1/2 and P-ERK1/2 declined compared with the control. 1) control group; 2) 5:2 (plasmid: reagent); 3) 6:2 (plasmid: reagent). **b**: ERK1/2 phosphorylation gradually declined as the concentrations of the inhibitor U0126, PD98059 increased, but total ERK1/2 protein expression hardly changed. 1) 0 μmol/L; 2) 5 μmol/L; 3) 10 μmol/L; 4) 20 μmol/L; 5) 40 μmol/L. **c**: ERK1/2 phosphorylation gradually declined as the concentrations of the inhibitor APDC. 1) 0 μmol/L; 2) 10 μmol/L; 3) 20 μmol/L; 4) 40 μmol/L; 5) 80 μmol/L. **d**: PD98059 can obviously inhibit the MEK1/2 phosphorylation level, but it did not alter MEK1/2 or NF-κB expression levels. 1) 0 μmol/L; 2) 5 μmol/L; 3) 10 μmol/L; 4) 20 μmol/L; 5) 40 μmol/L.

To investigate the effect of ERK1/2 expression and phosphorylation on DcR3 expression, BGC823 cells were treated with ERK1/2 shRNA or with inhibitors that specifically regulate the ERK pathway. Western blot analysis confirmed that the inhibitors efficiently blocked the phosphorylation of the MEK/ERK pathway molecules, and that the shRNA significantly reduced the expression of ERK1/2 (Figure 5). As shown in Figure 5A, when BGC823 cells were treated with ERK1/2 shRNA, ERK1/2 and P-ERK1/2 levels declined compared with the control.

U0126 is a very effective MEK inhibitor, resulting in the inhibition of ERK phosphorylation, as does PD98059. ERK phosphorylation gradually declined as the concentrations of the drugs increased, although total ERK1/2 protein expression hardly changed (Figure 5B).

APDC can inhibit NF-κB cell activation in a variety of cells. Through the degradation of IκB, APDC can decrease the translocation of NF-κB, thus blocking NF-κB activation. As shown in Figure 5 C, under different concentrations of APDC, changing the level of NF-κB inhibition can significantly attenuate ERK1/2 phosphorylation levels. However, the specific mechanism requires further investigation.

**Figure 6:**
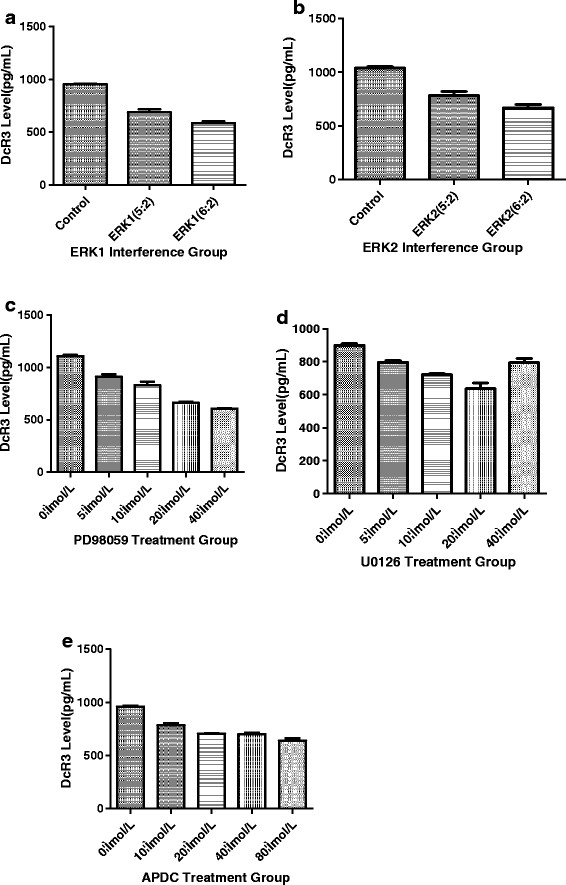
**The expression of DcR3 protein with plasmid interference and inhibitor by ELISA analysis. a**: DcR3 expression levels in BGC823 culture supernate decreased when cells were treated with ERK1 interference plasmid. **b**: DcR3 expression levels decreased when cells were treated with ERK2 interference plasmid. **c**: DcR3 expression levels decreased when cells were treated with different concentrations of U0126, PD98059 and APDC μmol/L.

To examine the effect of these inhibitors and shRNA on DcR3 expression we used ELISA analysis, which demonstrated that secreted DcR3 in the supernatant decreased after the different treatments (Figure 6). Statistical analysis showed that DcR3 secretion levels were significantly different between the experiment groups and control groups (*P* < 0.05). As shown in Figure 6, interference with ERK1/2 in BGC823 cells led to decreased DcR3 protein expression compared with the control group. The trend matches the ERK expression level in Figure 5 and proves that the two are positively correlated. Furthermore, DcR3 and P-ERK expression levels decreased when cells were treated with different concentrations of U0126, PD98059 and APDC. This data indicates that secretion of DcR3 positively correlated with P-ERK1/2 expression levels in BGC823 gastric cells. It is worth noting that in the U0126 group, DcR3 secretion levels increased when the drug concentration reached 40 μmol/L; however, the specific mechanism requires further investigation. In the APDC group, DcR3 levels did not change significantly at concentrations higher than 20 μmol/L.

## Discussion

It has been demonstrated that the DcR3 gene is expressed at a low level in human embryo, lung, brain, liver, spleen, stomach, colon, lymph nodes and spinal cord, whereas it was expressed at a high level in cancers such as gastrointestinal cancer, hepatocellular carcinoma and pancreatic cancer [1,12].

Wu *et al.*[8] reported that the expression of DcR3 in gastric cancer patients was significantly higher than normal. DcR3 expression in the well-differentiated gastric cancer was significantly lower than that of poorly differentiated specimens (*P* < 0.05).

The DcR3 expression level was significantly associated with lymph node metastasis and pathological stage, but did not correlate with tumor size, metastatic status, or histological types. When patients were followed-up for 63 months, DcR3 overexpression was found to be associated with a significantly shortened survival rate [13,14].

Many reports have shown that high expression levels of ERK1/2 closely correlated with breast, colorectal and pancreatic cancer, as well as malignant melanoma, leukemia and myxoma [15-18].

Our research showed that in patients with gastric cancer, the positive incidence of DcR3 and ERK1/2 mRNA was higher than that in the non-cancerous tissues (P < 0.05). RT-PCR and western blotting showed that the mRNA and protein expression levels of DcR3 and ERK1/2 in tumor tissues were significantly higher than those in non-cancer tissues, suggesting that DcR3 and ERK1/2 levels correlate with tumor development but not with age, gender or differentiation (*P* > 0.05).

Our results showed that the positive incidence of expression of DcR3 and ERK1/2 mRNA and DcR3 and ERK1/2 protein matched each other. The immunohistochemistry results also demonstrated that in gastric tumors, ERK1/2 was highly expressed.

Chen *et al.*[19] reported that the positive rate of DcR3 expression was 74.4% (32/43) in hepatocellular carcinoma, and that there was a significant correlation between DcR3 expression and metastasis as well as recurrence and differentiation.

In the mouse gastric model, RT-PCR showed that DcR3 mRNA could be detected in tumors from day six. ERK1/2 mRNA and protein were also detected in tumors, and ERK1 levels gradually increased in gastric tumors. Furthermore, they were also detected in heart, liver, spleen, lung and kidney of the gastric cancer animal model, suggesting that they have an important role in tumor progression. ERK1/2 mRNA was detected from day four in tumor tissues, and ERK1 mRNA peaked on day 10. ERK1 protein could also be detected on day four in tumor tissues, and continued to increase every day. ERK1 remained at a stable level in heart, liver and kidney, but it decreased in lung and spleen on day 10, after reaching the peak. ERK2 was detected on day two in spleen and tumor tissues. From day four, ERK2 was detected in all six tissues, and continued to increase till day 12. ERK2 could not be detected in heart, lung and spleen by RT-PCR on day 12 in animal models, but protein levels could be detected. These results suggest that ERK1 and ERK2 might have different effects on tumor occurrence, development and clonal expansion. Many studies indicated that the expression of ERK1/2 mRNA and protein varies in different tumors and cells [20,21]. Some recent reports suggested they could be entirely opposite in some cases. Thus, the effects of ERK1 and ERK2 on the tumors are unlikely to be the same.

## Conclusions

In conclusion, high expression of DcR3 and ERK1/2 may suppress tumor cell apoptosis and play an influential role in gastric cancer occurrence and development, which is an important mechanism in tumorigenesis [22]. In our study, DcR3 and ERK1/2 presented an overexpression tendency, and participated in the tumor immunity. We infer that in the tumor occurrence and developing process, the expression of ERK1/2 and DcR3 may be related to each other. Understanding the role of ERK1/2 in DcR3 expression might shed light on gastric cancer's diagnosis and identify a factor that regulates the expression of DcR3. It might be a new marker for early diagnosis of gastric cancer. DcR3 is a secreted protein, which can be detected in blood serum. Thus it could serve as a reliable index for clinical malignant tumor diagnosis, treatment and prognosis. Therefore, DcR3 has the potential of becoming a novel tumor marker in the future.

## Competing interest

The authors declare that they have no competing interest.

## Authors’ contributions

DY and XF carried out the molecular genetic studies, participated in the sequence alignment and drafted the manuscript. PY, QW carried out the immunoassays. FY, SY and BL participated in the sequence alignment. ZL participated in the design of the study and performed the statistical analysis. GZ conceived of the study, and participated in its design and coordination. All authors read and approved the final manuscript.
